# Perceptions and practices of community pharmacists towards the use of short-acting beta-2 agonists inhalers in Malaysia: A cross-sectional survey

**DOI:** 10.1371/journal.pone.0324982

**Published:** 2025-06-11

**Authors:** Zhe Chi Loh, Rabia Hussain, Bayan Faisal Ababneh, Jaya Muneswarao, Siew Chin Ong, Anees ur-Rehman, Zaheer-Ud-Din Babar

**Affiliations:** 1 School of Pharmaceutical Sciences, Universiti Sains Malaysia, Gelugor, Pulau Pinang, Malaysia; 2 Pharmacy Department, Hospital Pulau Pinang, George Town, Pulau Pinang, Malaysia; 3 Department of Pharmacy Practice, Faculty of Pharmacy, Bahauddin Zakariya University, Multan, Pakistan; 4 College of Pharmacy, Qatar University, Doha, Qatar; Isra University Faculty of Pharmacy, JORDAN

## Abstract

**Background:**

There has been a relative lack of exploration into the perceptions and practices regarding the use of SABA inhalers among healthcare professionals in Malaysia. The study aimed to determine community pharmacists’ perceptions and practices towards using SABA inhalers at community pharmacies in Malaysia.

**Methods:**

It was a cross-sectional study conducted using a self-administered, web-based survey (Google Form) among community pharmacists in Malaysia between 26^th^ December 2022 and 25^th^ May 2023. Descriptive statistics were used to summarize the participants’ socio-demographic characteristics, and the Kruskal-Wallis and Mann-Whitney U tests were applied to continuous data and p < 0.05 was considered significant. Regression analysis was carried out to identify associated predictors of socio-demographic characteristics of the participants regarding perception of asthma control and management.

**Results:**

A total of 312 community pharmacists completed the survey. Most of the participants were females (64.1%, n = 200), aged between 29 and 38 years (51.0%, n = 159). The majority of community pharmacists agreed that achieving good asthma control led to minimal asthma symptoms (n = 263, 84.3%) and fewer requirements for medical interventions (n = 204, 65.4%). Additionally, most participants were engaged in educating their patients about the correct techniques for using asthma inhalers (93.6%, n = 292) and provided sufficient information about the safe use of SABA inhalers (79.5%, n = 248). The most frequently perceived facilitator (n = 235, 75.4%) was providing additional follow-up sessions, and the primary perceived barrier (n = 232, 74.4%) was the lack of patients’ awareness of the provided asthma care services.

**Conclusion:**

Community pharmacists acknowledged that good asthma control was associated with minimal medical assistance and symptoms. However, some concerns were expressed over asthma patients’ purchase of non-prescription SABA inhalers. The participants perceived some barriers, such as lack of patient awareness regarding the asthma care services availability that might hinder the safe use of SABA inhalers.

## Introduction

Chronic obstructive pulmonary disease (COPD) and asthma are the most common chronic respiratory diseases [1]. COPD is a chronic lung condition of persistent airflow limitation commonly associated with acute exacerbations [2,3]. In 2019, COPD caused 3.23 million deaths worldwide, standing as the third leading cause of death globally [4]. Asthma is a chronic respiratory disorder characterized by airway inflammation and bronchoconstriction, affecting millions of individuals globally, contributing to significant morbidity and mortality [5]. In 2019, about 260 million people had poorly controlled asthma, and 461,000 deaths occurred due to asthma during the same year [6]. The cornerstone of asthma management is bronchodilation, with short-acting beta-2 agonists (SABA) representing a fundamental component in the treatment regimen [7]. SABA provides rapid relief from bronchoconstriction and is commonly prescribed both as rescue medication and as a pretreatment before exercise-induced bronchoconstriction [8]. SABA relievers are categorized under Group C items (medicines that pharmacists can dispense without a prescription) in Malaysia [9].

According to the literature, SABA overuse and SABA monotherapy have been linked to increased asthma exacerbations and asthma-related mortality [10,11]. Proper SABA use hinges on multiple factors, including adequate patient education, accurate diagnosis, and adherence to inhaled corticosteroids (ICS) [12]. The safe use of SABA requires attention, as individuals who overuse SABA often fail to acknowledge that frequent SABA usage may exacerbate their symptoms, and they may develop psychological reliance on SABA [13]. In line with this, the Global Initiative for Asthma (GINA) 2019 report recommended the as-needed low-dose ICS-formoterol as a preferred reliever for adult asthma patients, even in steps one and two of asthma treatments, whereas SABA-only treatment was no longer suggested [10,14]. Step 1 treatment is recommended for individuals experiencing symptoms less than twice per month without any significant risk factors for exacerbations, such as substantial environmental triggers, socioeconomic challenges, or severely impaired lung function [14]. On the other hand, step 2 treatment is intended for those patients who experience symptoms two or more times per month [14]. Also, the availability of SABA inhalers as over-the-counter (OTC) medicine has raised concerns regarding potential overuse [10]. According to a recent systematic review, 1.4% to 39.6% of OTC users worldwide were SABA inhaler users, and 14% to 66.4% of them were over users [15]. The risks of using OTC medicines included inaccurate self-diagnosis, reliance issues upon prolonged use, and adverse drug reactions [16]. Therefore, striking a balance between accessibility and responsible use through public education, pharmacist consultation, and clear usage guidelines is crucial to prevent the inadvertent escalation of SABA inhalers’ misuse due to increased availability [15,17,18].

The country-specific context of healthcare delivery and the practices of healthcare professionals can significantly impact patient care [15,16]. Like many other countries, Malaysia faces challenges in managing asthma effectively [19–22]. Despite the crucial roles that community pharmacists play in promoting appropriate SABA use and asthma management, the perceptions and practices of these healthcare professionals in Malaysia regarding the use of SABA inhalers remain relatively underexplored [16–19]. This study aimed to explore the community pharmacists’ perceptions and practices towards using SABA inhalers in the community pharmacy setting in Malaysia. Additionally, socio-demographic factors affecting the community pharmacists’ perceptions and practices towards using SABA inhalers in the community pharmacy setting were also explored.

## Methods

### Study design and setting

A descriptive cross-sectional study was conducted in Malaysia between 26^th^ December 2022 and 25^th^ May 2023. A combination of purposive and convenience sampling techniques was employed to collect data. These approaches aimed to encompass individuals with specific expertise while ensuring ease of accessibility [23]. A self-administered instrument using an online Google Form was utilized as a data-collecting tool, and was optimized as well to be easily filled while using computers and smartphones. It was then distributed by the authors to the community pharmacists through different social media platforms, including Facebook, WhatsApp, and Telegram applications, and emails. The time taken to complete the instrument was approximately 5–7 minutes. This study’s protocol was approved by the Human Research Ethics Committee of Universiti Sains Malaysia, namely Jawatankuasa Etika Penyelidikan Manusia (JEPeM) under the reference number of USM/JEPeM/22090575. Moreover, the participants who agreed to participate in the study, signed the consent form electronically before proceeding to the first section of the survey. The Google Form was optimized to receive one response from each participant. Additionally, the participation was voluntary and no incentives were provided for the participants.

### Participants

The sampling frame of the current study was 5271 community pharmacists in Malaysia [24]. The inclusion criteria were full-time community pharmacists registered with the Pharmacy Board of Malaysia, were proficient in English and provided informed consent to participate in this study. Part-time pharmacists, assistant pharmacists, and pharmacy technicians were excluded. A sample size of 312 community pharmacists was determined using the Cochran formula [25]:


$$n_{\circ }=\frac{Z^{2}pq}{e^{2}}$$

e: desired level of precision

p: estimated proportion of the population


q=1−p

### Study instrument

To assess the perceptions and practices of community pharmacists regarding the safe use of SABA inhalers, based on an extensive literature review, a closed-ended questionnaire (S1 Appendix) was designed, validated, and presented in English [13,15,26–29]. An evaluation team comprising four academics and field experts in clinical pharmacy and pharmacy practice at Universiti Sains Malaysia (USM) and Hospital Pulau Pinang evaluated the content validity of the questionnaire before distributing it to the participants. Any amendments were made based on the feedback and suggestions received. A content validity form was developed with a five-point Likert scale and emailed to the experts. They were requested to determine whether all items referred to the relevant aspects of constructs to be measured (1 = relevant, 5 = irrelevant), the importance of each item (1 = essential, 5 = not necessary), and any items missing in the questionnaire. After that, a pilot test using the online Google Form was performed among 40 community pharmacists who were selected conveniently from community pharmacies and excluded from the final analysis. The pilot test was conducted to identify and resolve the potential problems, such as identifying flaws, and deficiencies in the instrument [30]. Regarding face validity, participants provided insights on items’ degree of clarity (1 = the sentence is very vague, 5 = the sentence is very clear) and comprehension (1 = the sentence is tough to understand, 5 = the sentence is very easy to understand) by using a five-point Likert scale. To ensure the reliability of the instrument, internal consistency (which measures how closely related the items are for each scale) was calculated using Cronbach’s alpha coefficient, with values of 0.70 and above indicating good internal consistency [31].

The questionnaire was comprised of five sections, which were further divided based on 26 questions [32]. Participants’ demographics were obtained before the participants filled in the online survey. The first section consisted of six questions concerned with perceptions of asthma control and management, while the second part was comprised of seven questions that focused on risk perceptions of asthma reliever inhalers. The third section had four questions focused on practices regarding the use of SABA inhalers. The fourth and fifth sections of the survey each consisted of four and five questions, respectively, focused on facilitators and barriers to the safe use of SABA inhalers. A five-point Likert scale was used to measure responses: “strongly disagree,” “disagree,” “neutral,” “agree,” and “strongly agree”.

### Data analysis

Data analysis was conducted using IBM Statistical Package for the Social Sciences (SPSS)® v.20. Descriptive statistics were used to summarize the socio-demographic characteristics of participants. To assess the normality of the data, the Kolmogorov–Smirnov test was performed [33,34]. Since the data did not support parametric assumptions, the Mann-Whitney U test and the Kruskal-Wallis test were performed, as the Mann-Whitney U test was used to determine the differences between two groups of an independent variable, while the Kruskal-Wallis test determined the differences between more than two groups of an independent variable [35]. Categorical variable data were presented as frequency and percentage. The median and interquartile range were reported for continuous data. On the other hand, the overall perceptions and practices of community pharmacists were categorized by using Bloom’s cut-off point. Ratings fell into the ‘good’ range if the score ranged from 80 to 100%, ‘moderate’ if it spanned from 60 to 79%, and ‘poor’ if it was below 60% [36,37]. The total score was calculated by summing the item scores. The median total score, of 25, was used as the cutoff point for multiple logistic regression. Demographic characteristics were included as independent variables in the regression model. The findings of the regression analysis were presented as odds ratios (OR) with their corresponding 95% confidence intervals. A p-value of 0.05 was used for all statistical tests and was two-tailed. The Strengthening the Reporting of Observational Studies in Epidemiology (STROBE) checklist (S2 Appendix), which consisted of 22 items, was used for the reporting of this study.

## Results

### Demographic characteristics of the participants

The online survey response was collected until it reached the desired sample size of 312 participants. The majority of the participants were females (64.1%, n = 200), aged between 29 and 38 years old (51.0%, n = 159), Chinese (60.3%, n = 188), had a bachelor’s degree (95.8%, n = 299), had 5–10 years of work experience (44.9%, n = 140), and were from Penang (25.6%, n = 80), as shown in Table 1.

**Table 1:** Demographic characteristics of the participants (N = 312).

Variable	Frequency (n)	Percentage (%)
**Age**		
18-28 years old	93	29.8
29-38 years old	159	51.0
39-48 years old	29	9.3
49 years old and above	31	9.9
**Gender**		
Male	112	35.9
Female	200	64.1
**Ethnicity**		
Malay	95	30.4
Chinese	188	60.3
India	29	9.3
**Qualification**		
Bachelor’s degree	299	95.8
Master’s degree	13	4.2
**Working experience**		
Less than 5 years	110	35.2
5–10 years	140	44.9
10 years and above	62	19.9
**States of residing**		
Penang	80	25.6
Perak	17	5.5
Perlis	5	1.6
Sabah	9	2.9
Sarawak	13	4.2
Selangor	64	20.5
Terengganu	11	3.5
Kuala Lumpur	15	4.9
Labuan	1	0.3
Johor	41	13.1
Kedah	30	9.6
Kelantan	7	2.2
Melaka	12	3.9
Pahang	6	1.9
Negeri Sembilan	1	0.3

### Reliability and validity of the study instrument

The study instrument was amended according to the expert panel review. The Cronbach’s alpha coefficient for the instrument was 0.731, which showed that the data exhibited strong internal consistency in the format used.

#### Perceptions of asthma control and management.

Most of the community pharmacists (65.4%, n = 204) agreed that good asthma control was achieved when asthma could be managed with medical assistance, and when minimum asthma symptoms were present (84.3%, n = 263). The majority of participants expressed concerns (61.8%, n = 193) regarding the purchase of non-prescription SABA inhalers by asthma patients at community pharmacies who may not attend asthma reviews at hospitals. Additionally, 62.2% of the community pharmacists (n = 194) perceived that asthma patients would discuss their asthma-related concerns with community pharmacists.

**Fig 1:**
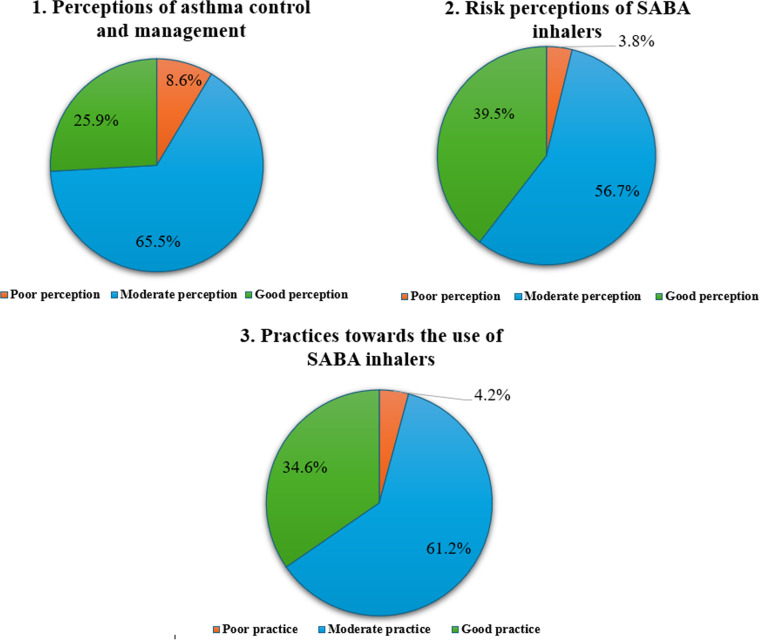
Graphical representation of Bloom’s cut-off points based on study data.

Further data analysis revealed that approximately 25.9% and 65.5% of participants had good and moderate perceptions of asthma control and management, respectively, with only 8.6% reporting poor perceptions (Fig 1). Notably, age was found to be significantly associated with the perception that good asthma control can be achieved with medical assistance (p = 0.005), the concern that asthma patients solely relying on SABA inhalers from community pharmacies may lead to poor asthma control (p = 0.036), and the positive outlook on asthma patients’ willingness to engage in discussions with community pharmacists (p = 0.037) (Table 2).

**Table 2:** Perceptions of asthma control and management (N = 312).

Statements	Participants’ responses, n (%)					Median (IQR)	p-value					
	SD	D	N	A	SA		Age^a^	Gender^b^	Ethnicity^a^	Academic qualification^b^	Working experience^a^	States of residence^a^
**1. Good asthma control is asthma that can be controlled with medical help.**	12 (3.8)	31 (10.0)	65 (20.8)	102 (32.7)	102 (32.7)	4 (2)	p = 0.005^*^	p = 0.521	p = 0.996	p = 0.143	p = 0.625	p = 0.500
**2. Good asthma control is having minimum asthma symptoms.**	12 (3.8)	8 (2.6)	29 (9.3)	86 (27.6)	177 (56.7)	5 (1)	p = 0.887	p = 0.840	p = 0.846	p = 0.511	p = 0.784	p = 0.500
**3. Do you perceive that it is reasonable for patients to change their asthma management if they really feel unwell on a particular day?**	24 (7.7)	43 (13.8)	97 (31.1)	89 (28.5)	59 (18.9)	3 (1)	p = 0.660	p = 0.166	p = 0.805	p = 0.533	p = 0.454	p = 0.039^*^
**4. Beside the medicines that were prescribed by physicians, do you think that patients managed their asthma with other over-the-counter (OTC) medicines?**	12 (3.8)	48 (15.4)	103 (33.0)	101 (32.4)	48 (15.4)	3 (1)	p = 0.387	p = 0.936	p = 0.065	p = 0.625	p = 0.299	p = 0.062
**5. Do you perceive that patients manage their asthma by decoction, herbal, or ayurvedic medicines?**	34 (10.9)	43 (13.8)	128 (41.0)	70 (22.4)	37 (11.9)	3 (1)	0.674	p = 0.159	p = 0.892	p = 0.636	p = 0.431	p = 0.297
**6. Asthma patients who always purchase their SABA inhalers from community pharmacies will lead to poor asthma control due to absence of asthma reviews at hospital.**	8 (2.6)	47 (15.1)	64 (20.5)	85 (27.2)	108 (34.6)	4 (2)	p = 0.036^*^	p = 0.297	p = 0.913	p = 0.790	p = 0.424	p = 0.198
**7. Asthma patients are willing to talk about their asthma problems to community pharmacists.**	19 (6.1)	51 (16.3)	48 (15.4)	131 (42.0)	63 (20.2)	4 (1)	p = 0.037^*^	p = 0.611	p = 0.142	p = 0.100	p = 0.182	p = 0.321

SD: Strongly disagree; D: Disagree; N: Neutral; A: Agree; SA: Strongly agree.

* Donates significant values (P-value<0.05).

NS: Non-significant associated.

^a^Kruskal Wallis test.

^b^Mann-Whitney U test.

### Risk perceptions of SABA inhalers

Most participants (69.9%, n = 218) perceived that asthma patients may develop an attachment to SABA inhalers. Additionally, around two-thirds of the participants, (74.3%, n = 232) perceived that asthma patients may experience side effects from using SABA inhalers.

In terms of risk perceptions related to SABA inhalers usage, it was found that 39.5% of participants exhibited scores greater than 80%, indicating good perceptions. Additionally, 56.7% scored between 60% to 79%, reflecting moderate perceptions, while only 3.8% scored below 60%, indicating poor perceptions (Fig 1). Furthermore, participants’ age and academic qualification revealed significant associations with two specific statements, as shown in Table 3. Firstly, there was a strong association between age and the perception that individuals using two or more puffs per day likely indicated uncontrolled asthma (p = 0.001). Secondly, participants’ age was also significantly linked to the belief that there is a high chance that asthma patients overuse SABA inhalers, which are requested from community pharmacies, compared with inhalers which are obtained from hospitals or clinics (p = 0.004). Moreover, there was a strong association between academic qualification and the perception that individuals using two or more puffs per day likely indicated uncontrolled asthma (p = 0.013). Lastly, there was an association between participants’ academic qualifications and the concerned perception about the frequencies and doses of asthma patients’ SABA usage compared with the techniques of utilizing SABA inhalers (p = 0.036).

**Table 3:** Risk perceptions of SABA inhalers (N = 312).

Statements	Participants’ responses, n (%)					Median (IQR)	p-value						
	SD	D	N	A	SA		Age^a^	Gender^b^	Ethnicity^a^		Academic qualification^b^	Working experience^a^	States of residence^a^
**1. Do you think that it is safe for patients to use more than two puffs of SABA inhalers per day?**	15 (4.8)	40 (12.8)	66 (21.2)	98 (31.4)	93 (29.8)	4 (2)	p = 0.465	p = 0.341	p = 0.563		p = 0.494	p = 0.703	p = 0.859
**2. If someone uses two or more puffs per day, most probably he/ she is having uncontrolled asthma.**	9 (2.9)	39 (12.5)	77 (24.7)	106 (34.0)	81 (25.9)	4 (2)	p = 0.001^*^	p = 0.185	p = 0.581		p = 0.013^*^	p = 0.552	p = 0.576
**3. Asthma patients should move to another inhaler completely which contained both a preventer and a reliever.**	12 (3.8)	17 (5.4)	73 (23.4)	102 (32.7)	108 (34.6)	4 (2)	p = 0.927	p = 0.538	p = 0.291		p = 0.497	p = 0.688	p = 0.002^*^
**4. Do you think that asthma patients develop attachment to SABA inhalers?**	0	23 (7.4)	71 (22.8)	93 (29.8)	125 (40.1)	4 (2)	p = 0.515	p = 0.210	p = 0.615		p = 0.660	p = 0.978	p = 0.510
**5. Asthma patients may experience side effects from the use of SABA inhalers.** **(For examples, dry mouth, palpitations, tremor, chest tightness, muscle cramps, headache)**	1 (0.3)	16 (5.1)	63 (20.2)	118 (37.8)	114 (36.5)	4 (2)	p = 0.383	p = 0.073	p = 0.420		p = 0.692	p = 0.435	p = 0.074
**6. There is a high chance that asthma patients overuse SABA inhalers which are purchased from community pharmacies compared with inhalers which are obtained from hospitals or clinics.**	5 (1.6)	27 (8.7)	66 (21.2)	79 (25.3)	135 (43.3)	4 (2)	p = 0.004^*^	p = 0.583		p = 0.314	p = 0.510	p = 0.082	p = 0.774
**7. Are you more concerned about the frequencies and doses of asthma patients’ SABA usage compared with the techniques to utilize the inhalers?**	47 (15.1)	58 (18.6)	80 (25.6)	70 (22.4)	57 (18.3)	3 (2)	p = 0.186	p = 0.142		p = 0.908	p = 0.036^*^	p = 0.324	p = 0.245

SD: Strongly disagree; D: Disagree; N: Neutral; A: Agree; SA: Strongly agree.

* Donates significant values (P-value<0.05).

NS: Non-significant associated.

^a^Kruskal Wallis test.

^b^Mann-Whitney U test.

### Practices towards the use of SABA inhalers

Most participants reported that they were involved in educating their patients on the correct techniques for using asthma inhalers (93.6%, n = 292) and provided sufficient information about the safe use of SABA inhalers (79.5%, n = 248). According to Fig 1, approximately 34.6% of participants exhibited good practices, 61.2% demonstrated moderate practices, and only 4.2% showed poor practices. According to Table 4, age was found to have a significant association (p = 0.009) with pharmacists’ practices regarding dispensing SABA inhalers to asthma patients when they perceived potential overuse. Conversely, a slight association (p = 0.048) was observed between the state of residence of the pharmacists, and their ability to provide adequate information on the safe use of SABA inhalers.

**Table 4:** Practices towards the use of SABA inhalers (N = 312).

Statements	Participants’ responses, n (%)					Median (IQR)	p-value					
	SD	D	N	A	SA		Age^a^	Gender^b^	Ethnicity^a^	Academic qualification^b^	Working experience^a^	States of residence^a^
**1. Pharmacists could dispense the SABA inhalers to asthma patients if they noticed that they might overuse it.**	35 (11.2)	51 (16.3)	106 (34)	78 (25)	42 (13.5)	3 (2)	p = 0.009^*^	p = 0.704	p = 0.135	p = 0.098	p = 0.114	p = 0.477
**2. Pharmacists could not dispense the SABA inhalers to asthma patients if they noticed that they might overuse it.**	32 (10.2)	59 (18.9)	137 (43.9)	42 (13.5)	42 (13.5)	3 (2)	p = 0.724	p = 0.662	p = 0.274	p = 0.432	p = 0.624	p = 0.068
**3. Have you ever educated the patients regarding the correct techniques to use asthma inhalers?**	3 (1)	5 (1.6)	12 (3.8)	124 (39.8)	168 (53.8)	5 (1)	p = 0.536	p = 0.691	p = 0.838	p = 0.211	p = 0.073	p = 0.158
**4. At my workplace, I believed that I provide enough information regarding the safe use of SABA inhalers to the patients.**	0	13 (4.2)	51 (16.3)	144 (46.2)	104 (33.3)	4 (1)	p = 0.492	p = 0.625	p = 0.937	p = 0.904	p = 0.167	p = 0.048^*^

SD: Strongly disagree; D: Disagree; N: Neutral; A: Agree; SA: Strongly agree.

* Donates significant values (P-value<0.05).

NS: Non-significant associated.

^a^Kruskal Wallis test.

^b^Mann-Whitney U test.

### Facilitators of the safe use of SABA inhalers

Offering more follow-up sessions (75.4%, n = 235) for asthma patients was identified as the most agreed facilitator for the safe use of SABA inhalers. The second most agreed facilitator among the participants (74.0%, n = 231) was the improvement of the duration of asthma counseling. Subsequently, 66.3% (n = 207) of the participants identified a monitoring system to track SABA inhalers purchases in community settings as a key facilitator, which was found to be significantly associated with gender (p = 0.011) and academic qualification (p = 0.047) (Table 5).

**Table 5:** Facilitators of the safe use of SABA inhalers (N = 312).

Statements	Participants’ responses, n (%)					Median (IQR)	p-value					
	SD	D	N	A	SA		Age^a^	Gender^b^	Ethnicity^a^	Academic qualification^b^	Working experience^a^	States of residence^a^
**1. A special room dedicated for asthma consultations.**	18 (5.8)	43 (13.8)	105 (33.6)	91 (29.2)	55 (17.6)	3 (1)	p = 0.059	p = 0.120	p = 0.272	p = 0.389	p = 0.154	p = 0.449
**2. Improve the duration of asthma counseling.**	6 (1.9)	12 (3.9)	63 (20.2)	133 (42.6)	98 (31.4)	4 (2)	p = 0.326	p = 0.172	p = 0.719	p = 0.802	p = 0.462	p = 0.767
**3. A monitoring system to keep track of the SABA inhalers purchasing records in the community setting.**	8 (2.6)	14 (4.5)	83 (26.6)	124 (39.7)	83 (26.6)	4 (2)	p = 0.296	p = 0.011^*^	p = 0.834	p = 0.047^*^	p = 0.559	p = 0.161
**4. Provide more follow up sections to the asthma patients.**	1 (0.3)	16 (5.1)	60 (19.2)	130 (41.7)	105 (33.7)	4 (1)	p = 0.747	p = 0.538	p = 0.521	p = 0.783	p = 0.300	p = 0.261

SD: Strongly disagree; D: Disagree; N: Neutral; A: Agree; SA: Strongly agree.

* Donates significant values (P-value<0.05).

NS: Non-significant associated.

^a^Kruskal Wallis test.

^b^Mann-Whitney U test.

### Barriers to the safe use of SABA inhalers

The majority of community pharmacist participants (74.4%, n = 232) perceived that the lack of patients’ awareness regarding asthma care services was a major barrier to the safe use of SABA inhalers. According to Table 6, this perception was found to be significantly associated with age (p = 0.037), academic qualification (p = 0.017), and state of residence (p = 0.025). Following this, lack of time was identified as the second most significant barrier (68.9%, n = 215) among the participants. The least perceived barrier (39.8%, n = 124) was the absence or insufficiency of incentives.

**Table 6:** Barriers to the safe use of SABA inhalers (N = 312).

Statements	Participants’ responses, n (%)					Median (IQR)	p-value					
	SD	D	N	A	SA		Age^a^	Gender^b^	Ethnicity^a^	Academic qualification^b^	Working experience^a^	States of residence^a^
**1. Lack of time.**	8 (2.6)	21 (6.7)	68 (21.8)	122 (39.1)	93 (29.8)	4 (2)	p = 0.910	p = 0.149	p = 0.591	p = 0.402	p = 0.511	p = 0.218
**2. Increased workload.**	21 (6.7)	16 (5.2)	97 (31.1)	99 (31.7)	79 (25.3)	4 (2)	p = 0.244	p = 0.095	p = 0.627	p = 0.660	p = 0.845	p = 0.830
**3. Less/ no incentives.**	42 (13.5)	52 (16.7)	94 (30.0)	71 (22.8)	53 (17.0)	3 (2)	p = 0.236	p = 0.260	p = 0.180	p = 0.162	p = 0.160	p = 0.210
**4. Lack of training.**	36 (11.5)	68 (21.8)	77 (24.7)	88 (28.2)	43 (13.8)	3 (2)	p = 0.418	p = 0.292	p = 0.937	p = 0.233	p = 0.315	p = 0.430
**5. Lack of patient awareness of the services.**	14 (4.5)	11 (3.5)	55 (17.6)	102 (32.7)	130 (41.7)	4 (2)	p = 0.037^*^	p = 0.592	p = 0.907	p = 0.017^*^	p = 0.706	p = 0.025^*^

SD: Strongly disagree; D: Disagree; N: Neutral; A: Agree; SA: Strongly agree.

* Donates significant values (P-value<0.05).

NS: Non-significant associated.

^a^Kruskal Wallis test.

^b^Mann-Whitney U test.

The logistic regression analysis was carried out to identify associated predictors of socio-demographic characteristics of the participants with perception of asthma control and management. According to Table 7, none of the variables showed a statistically significant association with perception of asthma control and management (p > 0.05).

**Table 7:** Factors associated with the participants’ perception of asthma control and management by logistic regression (N = 312).

Variable		OR (95%CI)	P-value
**Age**	**18-28**	Reference	
	**29-38**	0.84 (0.33-2.14)	0.713
	**39-48**	0.56 (0.14-2.19)	0.407
	**49 year and above**	0.50 (0.12-2.08)	0.344
**Gender**	**Female**	Reference	
	**Male**	1.01 (0.62-1.64)	0.967
**Ethnicity**	**Chinese**	Reference	
	**Malay**	1.11 (0.67-1.84)	0.693
	**Indian**	0.77 (0.34-1.76)	0.538
**Qualification**	**Bachelor’s degree**	Reference	
	**Master’s degree**	0.63 (0.19-2.07)	0.444
**Working experience (years)**	**Less than 5 years**	Reference	
	**5-10 years**	0.80 (0.33-1.93)	0.615
	**10 years and above**	1.41 (0.42-4.77)	0.578

## Discussion

This study provided an overview regarding the perceptions and practices of community pharmacists towards the safe use of non-prescription SABA inhalers among asthma patients in the community pharmacy setting of Malaysia.

Most of the community pharmacists (84.3%) agreed that good asthma control was achieved when minimum asthma symptoms were present. This concurs with the literature’s indication that people with well-controlled asthma would not need SABA inhalers and would have few or no asthma symptoms [38]. The majority of the participants of this study (62.2%) perceived that asthma patients would discuss their asthma-related concerns with community pharmacists on their own, suggesting the potential for pharmacist-patient engagement to promote good asthma control. According to a study, patients prefer pharmacists’ active involvement in asthma management, including asthma education, lung function tests, and monitoring [39]. In Saudi Arabia, nearly half (43.9%) of patients were satisfied with the counseling provided by community pharmacists [40]. In New Zealand, asthma patients experienced reduced reliance on SABA inhalers and increased adherence to ICS following interventions by community pharmacists, with most of them (78%) also reporting that the information provided by pharmacists was easy to understand [41].

In this study, most participants (68.6%) perceived that patients who consistently purchase non-prescription SABA inhalers may have a higher chance of developing uncontrolled asthma. Moreover, the majority of the community pharmacists (69.9%) stated that asthma patients may develop attachments to SABA inhalers and may experience side effects from using SABA inhalers (74.3%). Given this, community pharmacists, as the primary medical experts, play a crucial role as ‘gatekeepers’ in identifying potential medication safety issues, particularly concerning non-prescription medicines like SABA inhalers [15]. Additionally, various community pharmacist-led interventions have resulted in reduced reliance on SABA medicines and increased ICS adherence [41,42]. It has become evident that some asthma patients develop a psychological attachment to SABA inhalers due to their efficacy in rapidly alleviating asthma symptoms [13,15,28]. Considering the risks of frequent use of SABA inhalers, the GINA 2019 guidelines no longer recommend using SABA inhalers as monotherapy in the initial treatment of asthma [14]. Instead, using ICS and formoterol as preferred reliever options is encouraged [14]. Alternatively, a combination of a SABA and a low dose of ICS can be used, either in the same inhaler or separate inhalers, for symptom relief as the first step of asthma treatment in adults, even with mild asthma [14]. Notably, a recent meta-analysis indicated that when used within prescribed limits for symptom relief, the use of SABA as reliever therapy did not increase mortality or serious adverse events in adult asthma patients [7]. Therefore, community pharmacists’ understanding of the risks associated with frequent or improper use of non-prescription SABA inhalers can help prevent potential safety issues [15].

Community pharmacists should leverage their clinical expertise to identify potential overuse of SABA inhalers that were dispensed without prescription and engage patients in dialogue [43]. In this study, the majority of community pharmacists perceived that they had adequately provided education to their patients about the proper techniques for using SABA inhalers (93.5%) and had provided them with sufficient knowledge about asthma (79.5%). This concurs with a study done in Morocco and Saudi Arabia, where 75% and 85% of the community pharmacists respectively, reported that they teach their asthma patients about asthma and its management [44,45]. Community pharmacists may perceive themselves as effective educators and source of information for patients in managing their asthma [46]. However, patients may interpret or remember information differently, misunderstand instructions, or simply forget important details [46,47]. This scenario may be related to the low health literacy of certain asthma patients, for reasons such as inadequate reading, understanding, and comprehending medical information [48,49]. Several health literacy interventions such as Teach-Back, Simple Language, and Chunk and Check can bridge this gap [50]. The Teach-Back method entails patients repeating health information to ensure comprehension [48,51]. Utilizing Simple Language facilitates understanding by simplifying medical terminology [48,52]. The Chunk and Check strategy deconstructs complex information into smaller segments, aiding comprehension and confirmation [48].

Regarding the perceived facilitators for the safe use of SABA inhalers among the participants, the highly suggested facilitators were offering more follow-up sessions (75.4%) for asthma patients and improving the duration of asthma counseling (74.0%). Previous studies concurred with our findings that facilitators for the safe use of non-prescription SABA inhalers were frequent follow-up visits that were associated with regular updates and practical demonstrations, which were critical for effective asthma management [53]. In this case, community pharmacists can assess patients’ asthma control, monitor treatment compliance, and refer them to specialist medical consultants when necessary [54]. Sufficient counseling time has been shown to improve patient understanding and therapeutic outcomes, especially for those with complex treatment regimens, with sessions of around 30 minutes covering various aspects of asthma management [55,56]. However, challenges such as insufficient professional development and access to up-to-date medication information limit pharmacists’ ability to provide extensive counseling [55,57]. Additionally, 66.3% of participants suggested the need for a monitoring system to track purchases of SABA inhalers in the community setting, as evidence suggests a high tendency among asthma patients to overuse non-prescription SABA inhalers due to the lack of such a system [58]. Studies conducted in Australia indicated that about 73.9% of asthma patients purchasing non-prescription SABA were over-users, with 14% to 66.4% purchasing three or more SABA canisters OTC [26,27].

In this study, the most perceived barrier to promoting the safe use of SABA inhalers was a lack of patients’ awareness about the asthma care services offered by community pharmacists (74.4%). Nonetheless, the result of this study was aligned with the findings from other studies indicating patients’ limited awareness of community pharmacy-based asthma services and other services [39,59,60]. Some participants were unaware of the services offered by community pharmacists beyond their traditional dispensing roles [59,60]. However, upon learning about enhanced services, such as vaccinations and treatment advice for minor illnesses, they expressed interest in utilizing these options [59,60]. Enhancing patients’ understanding of specialized pharmacy services is crucial for service adoption and sustainability, leading to improved asthma outcomes in primary care [39]. Patients unaware of pharmacists’ asthma services may have lower expectations and express high satisfaction despite minimal services [39]. Additionally, community pharmacists’ constrained time may drive their preference for expedited dispensing; thus, having more technicians available to assist with dispensing duties would increase pharmacists’ time for asthma intervention [61–64]. On the other hand, the lack of incentives had been identified by 39.8% of the community pharmacists as a barrier to effective asthma management. Remuneration was crucial in motivating pharmacists to adopt a patient-centered approach to service delivery [65,66]. Notably, community pharmacists in France, Scotland, England, and certain states in the United States received remuneration for providing a range of asthma care services, including improving inhaler technique, offering asthma-enhanced services, providing education to patients, and monitoring the ongoing status of asthma patients [67,68].

### Study limitations

This is the first study to explore the perceptions and practices of community pharmacists concerning the use of non-prescription SABA inhalers by asthma patients in Malaysia. However, there were some limitations in this study that need to be acknowledged. The instrument for this study was distributed online, which targeted individuals with internet access, and responses from areas lacking such accessibility may not be captured. This may lead to demographic selection bias. Also, the majority of study participants were females and Chinese. Social desirability bias may still exist even though participant identities were kept confidential because they might have given answers they believed would be more socially acceptable rather than ones that really reflected their experiences or viewpoints. There were no right or wrong answers, and participants were guaranteed complete confidentiality and anonymity to minimize this bias. To prevent leading responses, the questions were written in neutral language, and no identifying information about the participants was connected to the collected data. Additionally, the instrument relied on participants’ self-rated assessment of their perceptions, which may have resulted in an overestimation of the results. Nevertheless, by encompassing participants from across Malaysia, this study remains a valuable source of insights into the perceptions and practices of community pharmacists regarding the use of SABA inhalers.

## Conclusion

Community pharmacists acknowledged that good asthma control was associated with minimal medical assistance and symptoms but expressed concerns over the purchase of non-prescription SABA inhalers by asthma patients. Most community pharmacists reported providing adequate education for asthma patients. They suggested raising awareness and educating asthma patients regarding risk perceptions and side effects associated with SABA inhalers. Additionally, the suggested facilitators among the community pharmacists for safe SABA inhaler use were increasing follow-up sessions and enhanced counseling, offering potential strategies for improving asthma care. Future studies are needed to explore the perceptions of other healthcare professionals towards the use of SABA inhalers at different healthcare settings in Malaysia.

## Supporting information

S1 Appendix Study questionnaire. (DOCX)

S2 Appendix The Strengthening the Reporting of Observational Studies in Epidemiology (STROBE) checklist. (DOC)

S3 Appendix Study data. (XLSX)
