# Correction: Perceptions and practices of community pharmacists towards the use of short-acting beta-2 agonists inhalers in Malaysia: A cross-sectional survey

**DOI:** 10.1371/journal.pone.0340305

**Published:** 2026-01-05

**Authors:** Zhe Chi Loh, Rabia Hussain, Bayan Faisal Ababneh, Jaya Muneswarao, Siew Chin Ong, Anees ur-Rehman, Zaheer-Ud-Din Babar

The Funding statement for this article is incorrect. The correct Funding statement is as follows: The study was supported by the Research and Innovation Division (RMCO) of Universiti Sains Malaysia through a Short Term Research Grant [reference number 304/PFARMASI/6315769].

## References

[1] Loh ZC, Hussain R, Ababneh BF, Muneswarao J, Ong SC, Ur-Rehman A, et al. Perceptions and practices of community pharmacists towards the use of short-acting beta-2 agonists inhalers in Malaysia: A cross-sectional survey. PLoS One. 2025;20(6):e0324982. doi: 10.1371/journal.pone.0324982 40498713 PMC12157093

