# Supplementary material for: Perceptions and practices of community pharmacists towards the use of short-acting beta-2 agonists inhalers in Malaysia: A cross-sectional survey
Source: PLoS One. 2025 Jun 11;20(6):e0324982. doi: 10.1371/journal.pone.0324982 (PMC12157093; doi:10.1371/journal.pone.0324982)
Supplement: S1 Appendix — (DOCX) [file pone.0324982.s001.docx]

Appendix 1: Perceptions and practices of community pharmacists towards the use of short-acting beta-2 agonists inhalers in Malaysia: A cross-sectional survey

(Questionnaire)

| In this section, we are interested to know about your perceptions regarding patients’ asthma control and management. Please indicate how much you agree with each of the following statements.  1= Strongly disagree, 2 = Disagree, 3 = Neutral, 4 = Agree, 5 = Strongly agree | | | | | |
| --- | --- | --- | --- | --- | --- |
| **Focus 1: Perceptions of asthma control and management** | | | | | |
| 1. According to you, good asthma control is: | | | | | |
| 1. Asthma that can be controlled with medical help. | ◯  1 | ◯  2 | ◯  3 | ◯  4 | ◯  5 |
| 1. Having minimum asthma symptoms. | ◯  1 | ◯  2 | ◯  3 | ◯  4 | ◯  5 |
| 1. Do you perceive that it is reasonable for patients to change their asthma management if they really feel unwell on a particular day? | ◯  1 | ◯  2 | ◯  3 | ◯  4 | ◯  5 |
| 1. Beside the medicines that were prescribed by physicians, do you think that patients managed their asthma with other over the counter (OTC) medicines? | ◯  1 | ◯  2 | ◯  3 | ◯  4 | ◯  5 |
| 1. Do you perceive that patients manage their asthma by decoction, herbal, or ayurvedic medicines? | ◯  1 | ◯  2 | ◯  3 | ◯  4 | ◯  5 |
| 1. Asthma patients who always requested their SABA inhalers from community pharmacies will lead to poor asthma control due to the absence of asthma review at hospitals. | ◯  1 | ◯  2 | ◯  3 | ◯  4 | ◯  5 |
| 1. Asthma patients are willing to talk about their asthma problems to community pharmacists. | ◯  1 | ◯  2 | ◯  3 | ◯  4 | ◯  5 |

| In this part, we are interested to understand about your perceptions of risks involve in asthma relievers use. Please indicate how much you agree with each of the following statements.  1 = Strongly disagree, 2 = Disagree, 3 = Neutral, 4 = Agree, 5 = Strongly agree | | | | | |
| --- | --- | --- | --- | --- | --- |
| **Focus 2: Risk perceptions of asthma reliever inhalers** | | | | | |
| 1. Do you think that it is safe for patients to use more than 2 puffs of SABA inhalers per day? | ◯  1 | ◯  2 | ◯  3 | ◯  4 | ◯  5 |
| 1. If someone uses 2 or more puffs per day, most probably he / she is having uncontrolled asthma. | ◯  1 | ◯  2 | ◯  3 | ◯  4 | ◯  5 |
| 1. Asthma patients should move to another inhaler completely which contained both a preventer and a reliever. | ◯  1 | ◯  2 | ◯  3 | ◯  4 | ◯  5 |
| 1. Do you think that asthma patients develop attachment to SABA inhalers? | ◯  1 | ◯  2 | ◯  3 | ◯  4 | ◯  5 |
| 1. Asthma patients may experience side effects from the use of SABA inhalers.   (For examples, dry mouth, palpitations, tremor, chest tightness, muscle cramps, headache) | ◯  1 | ◯  2 | ◯  3 | ◯  4 | ◯  5 |
| 1. There is a high chance that asthma patients overuse SABA inhalers which are requested from community pharmacies compared with inhalers which are obtained from hospitals or clinics. | ◯  1 | ◯  2 | ◯  3 | ◯  4 | ◯  5 |
| 1. Are you more concerned about the frequencies and doses of asthma patients’ SABA usage compared with the techniques to utilize the inhalers? | ◯  1 | ◯  2 | ◯  3 | ◯  4 | ◯  5 |

| In this section, we are interested to know about the practices of community pharmacists towards SABA usage. Please indicate how much you agree with each of the following statements.  1 = Strongly disagree, 2 = Disagree, 3 = Neutral, 4 = Agree, 5 = Strongly agree | | | | | |
| --- | --- | --- | --- | --- | --- |
| **Focus 3: Practices towards the use of SABA inhalers** | | | | | |
| 1. Pharmacists could dispense the SABA inhalers to asthma patients if they noticed that they might overuse it. | ◯  1 | ◯  2 | ◯  3 | ◯  4 | ◯  5 |
| 1. Pharmacists could not dispense the SABA inhalers to asthma patients if they noticed that they might overuse it. | ◯  1 | ◯  2 | ◯  3 | ◯  4 | ◯  5 |
| 1. Have you ever educated the patients regarding the correct techniques to use asthma inhalers? | ◯  1 | ◯  2 | ◯  3 | ◯  4 | ◯  5 |
| 1. At my workplace, I believed that I provide enough information regarding the safe use of SABA inhalers to the patients. | ◯  1 | ◯  2 | ◯  3 | ◯  4 | ◯  5 |

| In this part, we are interested to identify the possible factors that contribute to the safe use of SABA inhalers. Please indicate how much you agree with each of the following statements.  1 = Strongly disagree, 2 = Disagree, 3 = Neutral, 4 = Agree, 5 = Strongly agree | | | | | |
| --- | --- | --- | --- | --- | --- |
| **Focus 4: Facilitators to the safe use of SABA inhalers** | | | | | |
| 1. A special room dedicated for asthma consultations. | ◯  1 | ◯  2 | ◯  3 | ◯  4 | ◯  5 |
| 1. Improve the duration of asthma counselling. | ◯  1 | ◯  2 | ◯  3 | ◯  4 | ◯  5 |
| 1. A monitoring system to keep track of the SABA inhalers purchasing records in the community setting. | ◯  1 | ◯  2 | ◯  3 | ◯  4 | ◯  5 |
| 1. Provide more follow up sections to the asthma patients. | ◯  1 | ◯  2 | ◯  3 | ◯  4 | ◯  5 |

| In this part, we are interested to examine the possible factors that could hinder the safe use of SABA inhalers. Please indicate how much you agree with each of the following statements.  1 = Strongly disagree, 2 = Disagree, 3 = Neutral, 4 = Agree, 5 = Strongly agree | | | | | |
| --- | --- | --- | --- | --- | --- |
| **Focus 5: Barriers to the safe use of SABA inhalers** | | | | | |
| 1. Lack of time. | ◯  1 | ◯  2 | ◯  3 | ◯  4 | ◯  5 |
| 1. Increased workload. | ◯  1 | ◯  2 | ◯  3 | ◯  4 | ◯  5 |
| 1. Less / no incentives. | ◯  1 | ◯  2 | ◯  3 | ◯  4 | ◯  5 |
| 1. Lack of training. | ◯  1 | ◯  2 | ◯  3 | ◯  4 | ◯  5 |
| 1. Lack of patient awareness of the services. | ◯  1 | ◯  2 | ◯  3 | ◯  4 | ◯  5 |

This is the end of the questionnaires

Thanks to your contribution to the pharmacy research of Malaysia!
